# Libido–sexual disorders and abandonment of injectable contraceptives among users of the Ivorian Association for Family Well-Being in Korhogo, Côte d’Ivoire

**DOI:** 10.3389/fgwh.2023.1026253

**Published:** 2023-05-19

**Authors:** Esme Marie-Laure Essis, Katienin Jeanne Yeo, Djedou Martin Amalaman, Loukou Leandres Konan, Iba Bamba, Koné S. Aminata Coulibaly, D. Olga Denise Kpebo, Tetchi Orsot, Joseph Delafosse, Joseph Aka

**Affiliations:** ^1^ Center for Population and Health Policy and Systems Research, National Institute of Public Health, Abidjan, Côte d'Ivoire; ^2^ Reproductive Health Research Unit of Cote d'Ivoire, Abidjan, Côte d'Ivoire; ^3^ Department of Sociology and Anthropology, Peleforo Gon COULIBALY University of Korhogo, Korhogo, Côte d'Ivoire; ^4^ Department of Public Health and Biostatistics, Faculty of Medicine, Université Félix Houphouët Boigny, Abidjan, Côte d'Ivoire

**Keywords:** injectable contraceptive, libido-sexual disorders, adverse events, contraceptive abandonment, contraceptive, Family well-being, Korhogo, Cote d'Ivoire

## Abstract

**Introduction:**

The recent introduction of modern contraceptive methods in resource-limited countries is confronted with the occurrence of undesirable effects that hinder their use in the long term. This study conducted among the users of the Ivorian Association for Family Well-Being in Korhogo describes the libido–sexual problems associated with the discontinuation of injectable contraceptives in former users. The objective of the study was to identify the factors that led to the abandonment of injectable contraceptives among female users of the Ivorian Association for Family Well-Being in Korhogo between 2018 and 2019.

**Materials and methods:**

Qualitative data were collected from 15 former users (24–38 years old) of injectable contraceptives duration of 2–3 months. Additional data were collected from five health workers aged 35–60 years. In-depth interviews were conducted to explore the experience with injectable contraceptives and reasons for discontinuation. Following data collection, audio-recorded data were transcribed, translated, and coded using thematic analysis through an inductive approach.

**Results:**

Side effects identified as associated with injectable contraceptives include libido–sexual disorders, unusual bleeding, and weight gain. The most common reason for discontinuation were libido–sexual disorders, which impacted the households’ intimacy and provoked their abandonment or the change of contraceptive methods among injectable contraceptive users.

**Conclusion:**

Adverse events were dominated by libido–sexual disorders, unusual bleeding, and weight gain leading to the abandonment or change of the contraceptive. These results suggest points of intervention for increasing continuation among users. This intervention should include training of health workers to investigate and manage adverse events related to the use of injectable contraceptives and the improvement of communication between health workers and users on adverse events of injectable contraceptive use.

## Introduction

Modern contraceptive use remains low in many low- and middle-income countries despite strategies and policies to change behavior (1–3). This reality leads to an unmet need for family planning (FP), unwanted pregnancies, induced abortions, and high maternal-infant deaths (4–7). According to the literature, low contraceptive use is related to individual, sociocultural, legal, policy, and institutional factors that limit access to modern contraceptive methods (MCM) (1, 2). In addition, women's preferences for MCMs may be a barrier to contraceptive use. Several studies addressing FP and contraceptive use issues have highlighted women's preference for injectable contraceptives (1, 8, 9) as they ensure a discreet, easy, and nonrestrictive contraceptive practice. However, their practice is disrupted by the occurrence of adverse events in terms of side effects related to the use of these contraceptives. These are the most common reasons why users abandon them (10, 11). Several studies confirm that injectable contraceptives cause sexual and libido problems after a certain period of use in Europe (12–14), the United States (15–17), and Central Africa (18, 19). Although injectable contraceptive use remains low in sub-Saharan Africa with a slowly increasing tendency (11, 20, 21), side effects are already reported in this recent practice (18, 22). It is the case of the Ivorian context that is marked by a low national contraceptive prevalence (13.9%) with important regional disparities; a high maternal mortality (614 deaths per 100,000 live births) while 38 deaths per 1,000 live births are deplored in neonatology and 108 deaths per 1,000 live births in children under 5 years of age (23)*.* In response to this reality, the National Health Development Plan (2012–2015) has made the promotion of FP a priority, with a view to reducing maternal, infant, and neonatal mortality to improve socioeconomic development indicators in Côte d'Ivoire. It was noted that women are increasingly resorting to MCMs to limit births. These are pills (5.9%) and condoms (4.7%). The particularities of contraceptive use are noted in the northern region with a preference for injectable contraceptives, whose national use rate was estimated at 1.9% (DHS 2012). However, the long-term use of injectable contraceptives by women remains a concern for the managers of the Association Ivoirienne pour le Bien-Être Familial (AIBEF) in the city of Korhogo, an agency that provides support and advice to women who use MCMs. Because important cases of “lost to follow up” are noticed after about six months of users' follow-up. For example, the number of new users fell from 1,547 in 2018 to 701 in 2019 and 336 in 2020, with 76 cases of abandonment in 2018 and 57 cases in 2019, according to AIBEF monitoring reports. This steady decline in new injectable contraceptive adherence and dropout rates prompted an interest in documenting the problems with injectable contraceptive use among AIBEF Korhogo users, as of 2018, and in identifying key factors for dropout. While sexual and libido disorders are widely and extensively documented in developed countries (15, 16, 23), few studies, however, have focused on this topic in the West African context and, in particular, in Côte d'Ivoire. Hence, the present study was conducted with the aim of filling the scientific void in this area. It describes sexual and libido disorders as factors in the abandonment of injectable contraceptives among AIBEF users in Korhogo, Côte d'Ivoire.

## Material and methods

### Framework of the study

**Figure 1:**
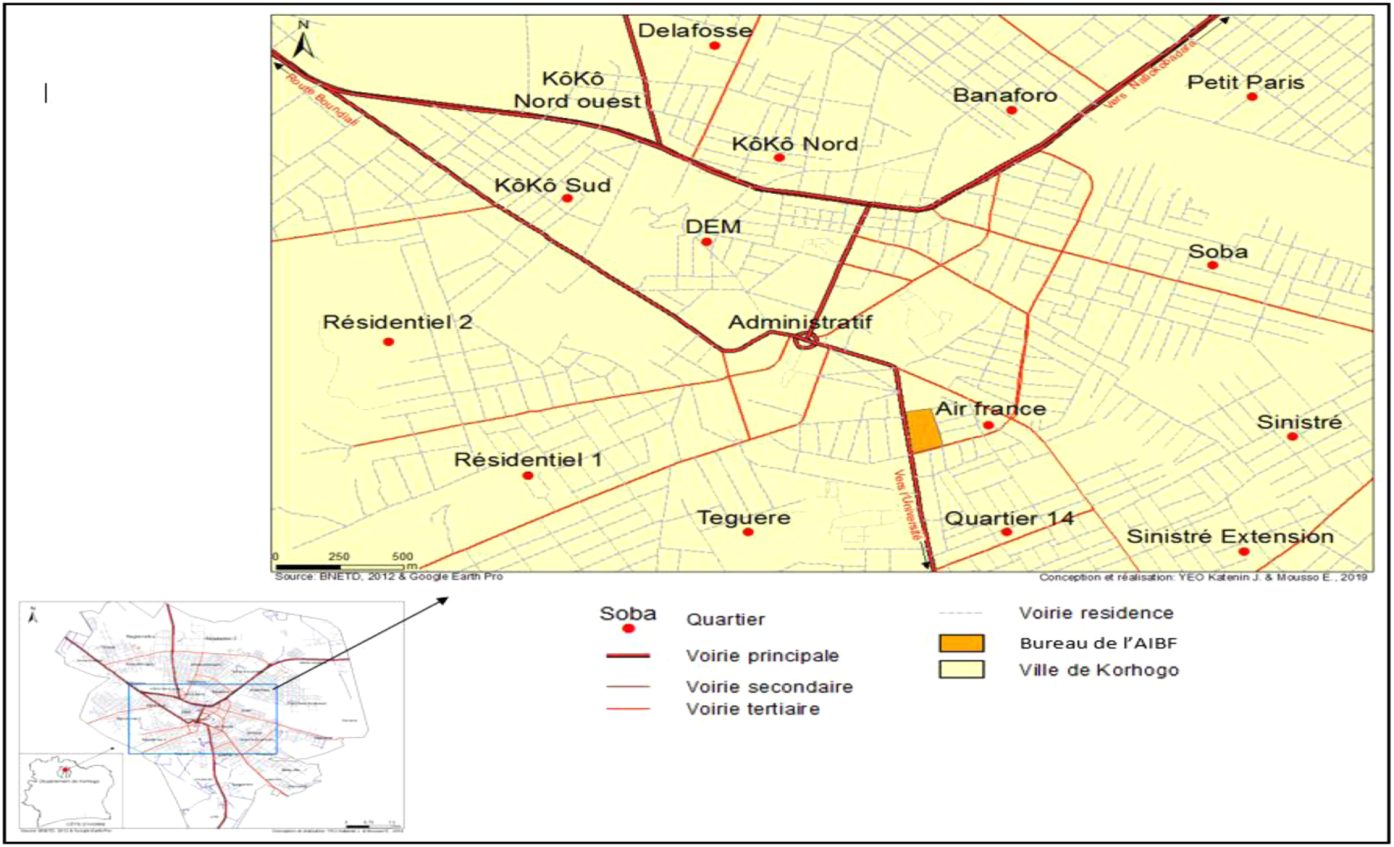
Location map of AIBEF in Korhogo.

Our study took place in the locality of Korhogo and within the AIBEF of this city. Korhogo is the fourth most populous city in Côte d'Ivoire, and the largest city in the north of the country. It is located 635 km from Abidjan and is the capital of the Savanes district and the Poro region, a strategic crossing point to Mali and Burkina Faso. The “City of Poro” covers an area of 12,500 km² for a population of 536,851 inhabitants (including 286,071 inhabitants for the commune of Korhogo), with 91.55% nationals and 8.45% non-nationals (RGPH 2014). It has a Sudanese tropical climate, and its main activities are agriculture, livestock, and trade (Figure 1).

### Study design and data collection tools

We conducted a qualitative study in which data were collected by direct interview using an interview guide addressing the following thematic areas:

• Reasons for the choice of injectable contraceptives by AIBEF users.
• Factors related to the abandonment of injectable contraceptives by these users.
• The undesirable effects that could slow down the use of injectable contraceptives.
• The wishes and suggestions of stakeholders for better use of injectable contraceptives in Korhogo.

### Sampling

The process of identifying respondents was done with combined “snowball” and “network” techniques. These techniques allowed us to go from one user to another after we contacted the head of the AIBEF health center in Korhogo and interviewed some of its staff. The AIBEF archives were also used to identify patients who had abandoned their contraceptive methods. This allowed us to identify their contacts and to approach former injectable contraceptive users who had switched to another method or who were no longer using contraceptive methods.

### Recruitment of study participants

Study data were collected from users and health workers at the AIBEF in Korhogo. The selection of participants was done to a maximum. Users were selected based on their experience of using injectable contraceptives. Meanwhile, the selection of health workers was based on their involvement in sexual and reproductive health services. We conducted the survey with 5health workers, namely, the coordinator, two providers (midwives), the FP educator, and the person in charge of logistics, and 15 former users of injectable contraceptives of AIBEF. These selections based on various files helped strengthen the data quality.

### Data collection

The study was carried out in two phases. The first phase concerned the pre-survey, which played an important role in the study, as it made it possible to discover and explore the field of investigation. We visited the health district and the AIBEF in Korhogo. We conducted some interviews with the head of AIBEF and their computer specialist. These interviews focused on the issue of contraceptive methods within their structure. In practical terms, the pre-survey allowed us to learn about the different types of methods available in their center and to get a feel for some of the realities in the field. This phase lasted for 5 days, from October 20 to 24, 2020, at the AIBEF center.

The second phase involved the actual survey. It took place over 3 weeks and 4 days, from November 5, 2020, to January 1, 2021, in the city of Korhogo. These two phases made it possible to meet with the various participants who made up our sample. The interviews were conducted with former users of injectable contraceptives and the health workers of the structure. We considered the availability of the various respondents (injectable contraceptive users and health workers) to talk with them outside of their work hours and on weekends. Data were collected by an experienced and trained sociologist (YKJ). All individual interviews were recorded on a Dictaphone with a built-in microphone and transcribed in French.

### Data analysis

The qualitative data collected and entirely transcribed in French by the members of the research team were entered and coded using Microsoft Word and Excel Windows 16.

After the transcriptions, a content analysis of the data allowed us to code the information collected and process it. To do this, we listed the information collected, the words recorded directly in audio. We noted the dialogs of the interviewees word by word, without modifying the content of the speech. The data are presented in the form of life stories and opinions that were used to learn about, identify, describe, expose, and classify in detail the factors related to the abandonment of injectable contraceptives among the users of the AIBEF of Korhogo.

### Ethical considerations

The study protocol was approved by the internal scientific committee of the National Institute of Public Health in Abidjan. Verbal and informed consent was obtained from the respondents before the interviews were conducted. The questionnaire was administered only when consent was obtained. Anonymity and confidentiality were respected; initials were used instead of names. Interviews were conducted in French or local language and in private locations to ensure confidentiality. In addition, permission was obtained from AIBEF and the Korhogo departmental and regional health directorate.

## Results

The 20 people in our sample who were interviewed included 5 health workers (2 women and 3 men) aged 35–60 years and 15 former injectable contraceptive users aged 24–38 years from the Ivorian Association for Family Well-Being (AIBEF) in Korhogo. In total, 17 women (85%) and 3 men (15%) were interviewed. The women used the injectable contraceptive (Depo-Provera) by intramuscular route with an action duration of 2–3 months, between 2018 and 2019. Our results are presented according to the following plan: (i) factors inherent in contraceptives that impact users’ sex lives and (ii) sexual and libido disorders inherent to contraceptives.

### Factors inherent in contraceptives that impact users’ sex lives

#### Overweight

The side effects of injectable contraceptive use on a woman's body are bothersome and annoying to users in all forms. Sudden weight gain can have a negative impact on their sex life and their relationship. It can then lead the user to abandon the contraceptive, according to the words of S.S., 28 years old, in training as a nurse's aide, living in an open relationship and mother of one child:

*“Personally, I didn't make that weight. I was on the two-month injectables, and I was fine with that because every eighty days I had my period. But since the two-month injectable went out of stock, I started taking the three-month injectable and I started to gain weight, from eighty kilos, I went to one hundred kilos in less than six months. Not all men can handle that, so I gave up the injection and went on the pill. Now I must pay for weight loss teas, medications to release all the hormones in my body.”*

This idea is echoed by K.A., an AIBEF provider, 45–50 years old, married with children, who talks about the inherent overweight of injectable contraceptives with these words:

“*In fact, the the dropouts are due to side effects. Because weight gain under the injectable contraceptive often creates problems. So, it causes many women to give up … This morning, I received a young girl who was put on injectable contraceptives at the Regional Hospital Center … Her mother finds that she is putting on too much weight, so she wants to use another method*.”

#### Bleeding

The injectable contraceptive is a synthetic progestogen with a high dose whose action consists of blocking ovulation and thickening the mucous membrane of the cervix for a variable period of 8–12 weeks. Blocking ovulation puts the woman's menstrual cycle at rest during the period of action of the progestin. This period is often disrupted by the appearance of unexplained bleeding with repercussions on the social and intimate life of women. They are indisposed to certain social activities and sexual relations, according to C.T., a 24-year-old student, single without children:

*“If you even hear that you are going to have sex, it means that your period has stopped. But it comes all the time. We're in the month of Lent and I can't pray, I can barely fast. So, I didn't have sex anymore because I was indisposed all the time, I had become very thin, I didn't reach fifty kilos (50 kg) anymore. I wonder if I even had sexual desires anymore … My only concern was how to stop my menstrual period*.”

Provider S.B., about 35–40 years old, married and mother, confirms the occurrence of cycle disorders related to injectable contraceptives and their consequences on the users’ experience:

*“When a woman is on contraception, it creates disorder in her couple. Because the fact that she sees her periods all the time makes the man indisposed and he can no longer have sexual relations at any time as he wants … When women use the injectable, they are indisposed all the time, which means that they cannot pray or perform their conjugal duty, so they are not at ease*.”

Amenorrhea secondary to the contraceptive also bothers the woman and constitutes, along with bleeding, a reason for abandonment, according to the words of the provider Y.P., between 50 and 55 years old, married with children:

“*Women often complain of amenorrhea or hypermenorrhea lasting more than ten days or even a month…or embarrassing spotting. Menstruation may come during the day and then stop, only to return in three days. Women come to complain repeatedly of amenorrhea and bleeding outside of menstruation*.”

### Sexual and libido disorders inherent to contraceptives

Most women surveyed reported sexual dysfunction and loss of libido after taking the injectable contraceptive. They admitted that they no longer felt sexual desire or pleasure during their various sexual relationships. S.F., a 29-year-old shopkeeper, married and mother of two children, confirmed these disorders:

*“When I took the injection, I was no longer aroused, I felt no pleasure, I no longer came during sex. There is only pleasure when you come or when you are excited. But I didn't feel any of that, so it's not interesting anymore*.”

S.S., a 28-year-old user in training to be a nurse's aide, living in an open relationship and mother of one child, agrees with the previous speaker and confirms the link with the abandonment of the contraceptive in these terms:

*“Also, I don't like sex very much, but the injection of the contraceptive made everything worse. It doesn't make me feel like having sex and it has taken me further away from sex. So, I often have sex like this, with no desire. But my partner feels like it, so I must touch him, touch him because he feels like it, how am I going to do it? when he's on top of me, I don't feel anything, I'm in a hurry for him to do it quickly and then leave, I think it's even lasting too long. When the person is not there, you think about him, but when he is there in front of you, it makes you angry. So, my darling says to stop the contraceptive because he doesn't feel his wife anymore oh*.”

These side effects of taking the injectable contraceptive are perceived by the spouses as negligence or even contempt or lack of interest in them by the woman. This situation creates conflicts that drive the spouses apart in a climate of jealousy and suspicion. This domestic crisis threatens the survival of the couple and leads women to abandon contraception to preserve harmony and peace. According to the experience of S.F., a 29-year-old trader, married and mother of two children, the use of injectable contraceptives has not been sexually beneficial, according to her:

*“My partner was reluctant to have sex when he saw that I was no longer interested in him, so he didn't ask for much. However, when I was not on injectable contraception, he always asked for it. But since I am on contraception, he has decreased, I tell myself that it is my attitude that caused this, because before it was myself who provoked him and brought him to do the sexual act, but when I started to take the injection there, it did not give me desire anymore. I was no longer interested in him, so he didn't ask me anymore*.”

S.F., a 29-year-old shopkeeper, married and mother of two children, underlines the marital conflicts that users of injectable contraceptives face in their households by saying:

*“My husband says he didn't know I could go a month without being interested in him. He insults me by accusing me of cheating on him with other men when I go to sell*.”

The remarks of S.S., a 28-year-old nurse's aide trainee, living in a common-law relationship and mother of one child, support this:

*“It almost caused a fuss between me and my boyfriend. He told me that the injectable contraceptive is a drug that makes you lose your sexual desire. The wife of one of his friends who was taking it had lost all sexual desire to the point that his friend thought that his wife was cheating on him with other men because she was pushing him away at every attempt.”*

In view of these remarks, we can easily understand what users of injectable contraceptives experience and feel. In addition to the effects on the woman's body and her cycle, libido–sexual problems have a lasting impact on the wellbeing of users. The lack of sexual appetite and the decrease in pleasure linked to the conjugal act have an impact on the quality of life of the woman. They are no longer able to fulfill their marital duties in a qualitative way, so they abandon the injectable contraceptive.

### Sexual arousal inherent in contraceptives

Some participants stated that the weight gain associated with the use of the injectable contraceptive was well appreciated by their partners and reawakened their sexual appetite, according to S.S., a 28-year-old nurse's aide, living in a common-law relationship and mother of one child:

*“My husband refuses to let me go out. Even when I am in front of the gate, we talk because there are boys in front of the yard. He says that I've become too pretty and that I've grown a bit of a bum. So, he doesn't want me to go to the market, he takes the food from his parents to send to me or he pays for food … and when he's not there, just a little bit he calls me, what are you doing? Are you lying down? What position did you take? Hey hey hey!!!*”

Through this report, we understand that the use of the contraceptive has been a source of motivation for sexual practice. Because the contraceptive gave users confidence, it contributed to a certain sexual wellbeing. In addition, contraceptive use has made sexual activity more recurrent among men, with some abuse. In fact, the woman on the contraceptive was more attractive because of the development of her curves when she gained weight. So, the spouses were more attached to their wives, and this increased the frequency of sexual intercourse. This reality was experienced by S.S., 28 years old, in training as a nurse's aide, living with a partner and mother of one child who goes further with this revelation of the effect of weight on the vagina:

*“Hmmm! It used to be better! when I went on birth control, it increased his cravings because when you get fat, your sex shrinks. So, he found his business!!!…..I tell him ah! my brother, I am also a child of people!!! Just a little, come I'll tell you something and then he takes me to have sex. We even palavered for often two or three days; he does not speak to me. He goes so far as to tell me that I'm doing sexual disobedience. If he was the only one there, every day he would have sex*.”

The contraceptive has impacted positively on the sex lives of some users because the security it provides has contributed to the sexual development of their couples. On this point, we recorded the opinions of users who perceived the use of the contraceptive as a positive element in their sexual life. C.N., a 31-year-old shopkeeper married with three children, said

*“When I took a shot there it made my case worse. I always asked my partner to make love to me, because I knew I was safe*.”

These comments were supported by those of S.K., 26 years old, student, single and without children:

*“In any case, it gave me the courage even to make love. Because it was when I wanted to start a boy's business that I went to take the injection so as not to get pregnant out of wedlock, or that would put an end to my studies, so I was not afraid, I was calm, and I was not afraid of anything anymore*.”

However, users have experienced a crisis of trust and tension in their relationships. Contraception gives freedom to the woman, while at the same time fomenting jealousy and doubts in the man about the fidelity and sincerity of his partner. For him, a woman on contraception can easily cheat on her man because she is safe from unwanted pregnancies. This is expressed by K.A., 45–50 years old, married and mother of a family, provider, through his words:

*“No man wants to see another man touch his wife. There is the case of a woman who was at my home here, her husband called him to know if she had arrived and if it was a man or a woman who was receiving her … I made him understand that in our home, no one has the right to touch another's wife and that we have two midwives who take care of the patients, so no one is going to touch his wife*.”

## Discussion

The present study, which describes the libido–sexual disorders related to the abandonment of the injectable contraceptive in Senufo in Korhogo, is a first in Côte d'Ivoire. It raised two major questions that deserve to be discussed: (i) the sexual and libido disorders inherent to contraceptives that are the main subject of this study; (ii) the factors inherent to contraceptives that explain some of these described disorders.

### Sexual and libido disorders inherent to contraceptives

The main results of the study show that the side effects that impact the intimacy of the household and the libido–sexual disorders that cause tension in couples justify abandoning the injectable contraceptive or changing the method. These results are similar to those of previous studies that have looked at the sexual function of hormonal contraceptive users (12, 15, 17, 24, 25).

The users of the injectable progestin were confronted with a decrease in their sexual desire; they did not feel any excitement during the sexual act, which they endured as a real chore. Libido–sexual disorders have an impact on a woman's reproductive health and the harmony of the couple. Sexual relations are part of the physiological needs of the individual, so that any dysfunction affects the health of the individual, with repercussions on his or her abilities and creativity, while compromising the procreative function (22, 25).

Our results also reported positive effects of the contraceptive on sexual function in terms of sexual arousal similar to other studies in the literature (15, 22). In general, the effects of injectable contraceptives on sexual function reported in the literature are controversial and confirm our own.

To date, there is no plausible explanation for the mixed results of hormonal contraceptives on the health of users. The results are different and often contradictory from one study to another. Indeed, for some authors, the impact of progestogen contraceptives on female sexual dysfunction remains minimal (16), while for others, satisfaction during sexual activity depends on factors beyond sexual functioning alone (26). Both et al. denounced a lack of sufficient evidence to establish a clear algorithm for managing hormonal contraceptive-induced sexual dysfunction due to controversies (27). Therefore, the multifaceted nature of female sexual function justifies the importance of establishing a temporal relationship between the onset of sexual complaints and the initiation of hormonal contraception. This is because several factors affecting sexual response may cover the positive or negative impact attributable to hormonal contraception (15). When in doubt, the same contraceptive that produces opposite effects at the same time is subject to discussion and reopens the old debate about the safety of contraceptive products on maternal health. The wellbeing of the woman and the couple sought using modern contraceptives is finally understood by the consequences of their adverse effects. Further studies are needed to reach accurate conclusions and make informed decisions.

However, our supposedly positive contraceptive outcomes have also led to crises of trust and tension in the couple and have caused them to abandon taking the contraceptive. Indeed, the sexual excitement and security of the contraceptive have created situations of sexual abuse that have eventually led to partner fatigue and revolt. Especially since this excitement is surrounded by a climate of unhealthy jealousy, which in the long run suffocates the partner and forces the woman to abandon the contraceptive (28).

### Factors inherent in contraceptives that impact users’ sex lives

Contraceptive-related bleeding makes the woman unavailable for her religious practice (going to the mosque to pray, practicing the Muslim fast, etc.), for certain social activities, and for her partner. In short, bleeding creates situations of discomfort for the woman and the man, generating tensions and crises in the household and affecting the intimate relationship, which leads to the abandonment of the progestin. This result is similar to that of Anoua Adou (2016), who links the side effects (bleeding, pain, amenorrhea, etc.) of the injectable contraceptive with the deterioration of social relations and the risk of the couple breaking up. These disadvantages have led to mistrust of the contraceptive and the abandonment of its use in the rural Ivorian environment of Gwa of Domlon in the health district of Alépé, in the southeast of the country. It was also noted that men's reluctance to use FP was related to the fear of losing control over women's fertility and sexuality (28).

The extra weight associated with contraception, although it bothers women, is well appreciated by men who find it beneficial. Indeed, Ivorians are fond of curves and look for women with generous shapes most of the time. This trend is due to a revelation of this study according to which weight gain shrinks the vagina of the woman and improves the pleasure associated with the sexual act. Weight gain and menstrual problems were the major side effects of injectable contraceptives and implants in the study by Bangoura et al. conducted in Guinea among adolescents and young urban users. The sexual disorder found was a low sensation of sexual pleasure, which was more related to the use of condoms (29).

The contraceptive injection, under the trade names Depo-Provera, Sayana Press, and Noristerat, is a long-acting progestin steroid (progesterone). It suppresses the production of follicle stimulating hormone (FSH) and inhibits the increase in estrogen levels. Its effect reduces serum estradiol levels and is closely linked to users’ complaints of mood changes, depression, and decreased sexual desire (17). It is contraindicated in some cases of illness and pregnancy, so careful medical supervision is required before and during its use. However, it should be noted that its use in developing countries, especially in Africa, is not always accompanied by a health check-up (28). Women are put on injectable contraceptives without any medical analysis to assess the non-noxiousness of the product. Everything is done blindly. In this context, it is difficult to objectively attribute the occurrence of an adverse effect to the contraceptive. In addition, the absence of a medical check-up creates a psychosis and develops myths and erroneous perceptions that amplify fear among users, pushing them to abandon the contraceptive at the appearance of any discomfort. In addition, health workers do not have the in-depth knowledge of the various contraceptive products to better advise women and manage adverse events (15, 26, 30).

## Study limitations

The difficulties encountered were related to data on patients no longer using injectable contraceptives. In fact, there were no data for each type of method, so we had to refer to the archives to find them. Some users were not contacted because their records were incomplete, and their contacts were not included. Another difficulty encountered was a lack of confidence in the confidentiality of their statements. This mistrust is justified by the fear of indiscretion felt by some users and health workers. In addition, our respondents found some questions difficult or too private. Some respondents wanted the interviews to be held outside their homes, even in the service, or in places where women gather or somewhere in town. The interviews that took place at the clinic were shorter because they were held during off-duty hours, and there were actors who gave us appointments that they did not keep.

## Conclusion

The results of this study were marked by the occurrence of bothersome side effects after 6 months of use of injectable contraceptives (*Depo-Provera, Sayana Press*, and *Noristerat*) at the AIBEF in Korhogo. These adverse events were dominated by libido–sexual disorders, unusual bleeding, and weight gain, which, while testing the harmony and intimacy of the couple, led to the abandonment or change of the contraceptive. This study shows the urgency of filling the gaps of health workers to better support contraceptive practice to obtain better results and reverse the curve of maternal and child mortality. Moreover, given the controversial results of modern contraceptives on maternal health, we recommend to:

– supervise the setting under MCM of medical examinations and analyses,
– investigate and manage adverse events related to taking MCM,
– train health workers on MCMs for better care of users,
– improve communication between health workers and users, and
– improve monitoring of MCM users, especially those with adverse events.

Prospective research with larger numbers of women is needed to improve understanding and document the potential libido–sexual side effects of injectable contraceptives on a national level.

## Data Availability

The raw data supporting the conclusions of this article will be made available by the authors, without undue reservation.
